# The ER-Golgi transport of influenza virus through NS1-Sec13 association during virus replication

**DOI:** 10.1128/spectrum.02609-23

**Published:** 2023-12-01

**Authors:** Sonja C. J. H. Chua, Jianzhou Cui, Karishma Sachaphibulkij, Isabelle Siang Ling Tan, Hui Qing Tan, Hong Meng Lim, David Engelberg, Lina H. K. Lim

**Affiliations:** 1 Department of Physiology, Yong Loo Lin School of Medicine, National University of Singapore, Singapore, Singapore; 2 NUS Immunology Program, Life Sciences Institute, National University of Singapore, Singapore, Singapore; 3 NUSMED Immunology Translational Research Programme, National University of Singapore, Singapore, Singapore; 4 CREATE-NUS-HUJ Molecular Mechanisms of Inflammatory Diseases Programme, National University of Singapore, Singapore, Singapore; 5 Department of Biological Chemistry, The Institute of Life Science, The Hebrew University of Jerusalem, Jerusalem, Israel; 6 Department of Microbiology, Yong Loo Lin School of Medicine, National University of Singapore, Singapore, Singapore; Technion - Israel Institute of Technology, Haifa, Israel

**Keywords:** influenza, H1N1, H3N2, non-structural protein 1 (NS1), Sec13, COPII

## Abstract

Influenza A virus is a respiratory virus that can cause complications such as acute bronchitis and secondary bacterial pneumonia. Drug therapies and vaccines are available against influenza, albeit limited by drug resistance and the non-universal vaccine administration. Hence there is a need for host-targeted therapies against influenza to provide an effective alternative therapeutic target. Sec13 was identified as a novel host interactor of influenza. As Sec13 is a member of the nuclear pore complex and coat protein complex II (COPII) vesicles, localization of both Sec13 and non-structural protein 1 (NS1) in the nucleus, endoplasmic reticulum (ER), COPII vesicles (ER-to-Golgi transport), and Golgi was studied during infection. Sec13 is associated with ER, COPII, and Golgi in infected lung epithelial cells and not the nucleus during PR8 infection. This observation would imply the functional role of Sec13 in the COPII vesicles (ER-to-Golgi transport). Moreover, the colocalization of NS1 and Sec13 were correlated at several time points of infection, indicating the function of Sec13 during influenza infection. Inhibiting the ER-to-Golgi transport and silencing Sec13 decreased viral titers, whereas overexpressing Sec13 increased viral titers. Hence, we propose that the ER-to-Golgi transport is an important pathway of viral replication and viral export, and specifically, Sec13 has a functional role in influenza replication and virulence.

**IMPORTANCE:**

Influenza A virus is a respiratory virus that can cause complications such as acute bronchitis and secondary bacterial pneumonia. Drug therapies and vaccines are available against influenza, albeit limited by drug resistance and the non-universal vaccine administration. Hence there is a need for host-targeted therapies against influenza to provide an effective alternative therapeutic target. Sec13 was identified as a novel host interactor of influenza. Endoplasmic reticulum-to-Golgi transport is an important pathway of influenza virus replication and viral export. Specifically, Sec13 has a functional role in influenza replication and virulence.

## INTRODUCTION

The influenza virus is a negative-sense RNA virus transmitted via respiratory droplets. It causes acute respiratory diseases in humans. Three to five million people are affected with severe symptoms every year, and 500,000 die from complications due to influenza (1). Young children, the elderly, and patients with chronic conditions like diabetes, heart disease, kidney disease, and immunocompromised are vulnerable to severe influenza (1, 2). Severe influenza may develop into complications such as secondary bacterial pneumonia and acute bronchitis (3).

The influenza genome consists of symmetrical helixes in eight segments numbered according to their decreasing lengths. The segments encode for surface glycoproteins (haemagglutinin [HA] and neuraminidase [NA]), matrix protein (M1), matrix ion channel (M2), viral RNA-dependent RNA polymerase complex (composed of nucleoprotein [NP], polymerase basic subunits [PB1 and PB2], and polymerase acid subunit [PA]), and two nuclear export proteins (non-structural protein 1 [NS1] and NEP/non-structural protein [NS2]) (4, 5). Due to viral adaptation and reassortment, highly virulent strains sporadically appear and cause local epidemics or global pandemics, such as the 1918 H1N1 Spanish pandemic, 2005 H5N1 Bird flu, and the 2009 H1N1 Swine flu (6).

Currently, only two antiviral drug classes with some usefulness are available, and very few are under development or trials (7). The drugs that have been approved for the treatment of influenza are M2 ion channel inhibitors (e.g., amantadine and rimantadine) and NA inhibitors (e.g., oseltamivir and zanamivir) (8). Drug-resistant virus strains have rendered M2 inhibitors ineffective, and there is growing resistance against NA inhibitors such as the H274Y mutation found in the 2009 H1N1 strain (6, 9). Newer drugs, such as favipiravir, a nucleotide analog that targets the viral polymerase, was approved in Japan, and baloxavir marboxil, which is a cap-dependent endonuclease inhibitor, has been approved in Japan and the USA (10), with pimodivir, a PB2 inhibitor, having pre-approval access by the FDA (NCT03834376). Even during phase 2 clinical trials, resistant viruses were selected for the treatment of baloxavir due to I38T substitution (11). The primary strategy to prevent or control seasonal influenza epidemics is the annual vaccination program. Due to its varied effectiveness year to year, pandemics and epidemics still emerge when vaccines are mismatched with the predominant antigenic strains (12).

Sec13 was first identified in yeast as Sec13p. It is a 36 kDa protein that consists of six repeated sequence motifs, known as WD-40 repeats (13). Sec13 is found in the nuclear pore complex (NPC) and COPII vesicles. When Sec13 was purified from yeast, most of it was copurified in a 700 kDa protein complex (13, 14). Upon further investigation, Sec13p is found to be associated with Sec31p, and this complex is part of the coat protein complex II (COPII), which is involved in the transport from the endoplasmic reticulum (ER) to the Golgi apparatus (14, 15). Evidence supported that these two proteins form a heterotetramer with two copies of each Sec13 and Sec31 (16). The Sec13/31 structure mainly comprises WD40 domains and α-solenoid motifs (17).

Sec13 has been identified as an essential host interactor in viral infections such as brome mosaic virus (BMV) in plants (18) and HIV (19). Perinuclear ER localization of BMV 1a is disrupted in cells expressing dysfunctional COPII coat components such as Sec13 (18). Furthermore, Sec13 depletion was found to inhibit HIV-1 infection by reducing HIV integration (19). This would highlight the key role Sec13 involved in viral replication which may extend into influenza. In addition, protein processing in the ER pathway was previously identified as one of the main processes of influenza interactors (5). Gathering the functional importance of Sec13 displayed in various key viruses (HIV and BMV), the replicative/extended role of Sec13 in the influenza remains poorly understood. Therefore, the role and function of Sec13 in the influenza life cycle and were investigated.

## MATERIALS AND METHODS

### Retrieval of NS1 and Sec13 crystal structures

The crystal structures of NS1 from various viruses were retrieved from Protein Data Bank (PDB). Their respective PDB IDs are listed in Table S1. The crystal structures of Sec13 were retrieved from the PDB with PDB IDs of 3BG0 (https://www.rcsb.org/structure/3BG0) and 3BG1 (https://www.rcsb.org/structure/3BG1).

### Protein–protein docking using ClusPro

ClusPro works in three main steps: First, it runs PIPER, a rigid body docking program based on a novel fast Fourier transform docking method with pairwise potentials. Second, the 1,000 best energy conformations are clustered, and the 30 most significant clusters are retained for refinement by using a clustering technique for the detection of near-native conformations and by eliminating some of the non-native clusters. Third, the stability of these clusters is analyzed by short Monte Carlo simulations, and the structures are refined by the medium-range optimization method—semi-definite programming-based underestimation (20).

### Cell culture

Human lung epithelial A549 cells were maintained in Dulbecco's modified Eagle medium (DMEM). The media was supplemented with 10% fetal bovine serum (FBS) and 1% penicillin–streptomycin. All cell lines were maintained in a 37°C humidified incubator receiving 5% CO_2_ (Thermo Fisher Scientific, Singapore).

### Transfection of cells

A549 cells were seeded 1.05 × 10^5^ cells in 500 µL growth medium for a single well of a 24-well plate for 8 h before transfection into tissue culture plates. The cell culture must be 80% confluent on the day of transfection. Transfection of A549 was performed with Turbofect (Thermo Fisher Scientific) according to manufacturer instructions.

### Sec13 silencing using DsiRNA knockdown

Predesigned Sec13 DsiRNA (dicer-substrate siRNA) (Cat# hs.Ri.Sec13.13.1;Sense:5′–rArGrArCrArGrGrUrCrCrGrUrArArArArUrCrUrUrUrGAT-3′, Antisense:5′–rArUrCrArArArGrArUrUrUrUrGrArCrGrGrArCrCrUrGrUrCrUrGrA-3′) was transfected into the A549 using a commercial TriFECTa RNAi kit (Integrated DNA Technologies, Singapore). Negative control (NC) DsiRNA from the TriFECTa RNAi kit (Cat# 51011403) was used as a NC. Cells were reverse transfected with 10 nM of DsiRNA and lipofectamine RNAIMAX transfection reagent (Thermo Fisher Scientific) overnight according to manufacturer's instructions. Knockdown efficiency was subsequently determined using western blot and quantitative PCR.

### Infection of cell culture with IAV

For infections involving A549 cells, 5 × 10^6^ A549 cells were seeded 24 h before infection in two T75 flasks. After 24 h, media was aspirated, and cells were washed twice with 1× PBS. Media without serum was added, and virus inoculum was added at specified multiplicity of infection (MOI). After 1 h of infection, cells were washed again with PBS, and complete DMEM was added for the indicated times for infections. The volume of A549 cells was adjusted so that the final concentration is 2 × 106 cells/mL. Virus strains used in this study were A/Puerto Rico/8/1934 (PR8) (H1N1) and A/Hong Kong/1/68(H3N2).

### Determination of virus titer using plaque assay

1.5 × 10^5^ Mardin–Darby canine kidney (MDCK) cells per well were seeded in a 24 well plate (Falcon, #353047) and allowed to grow overnight. The following day, cells were washed and infected with a 10-fold serial dilution of the virus in 1× PBS in a total of 250 µL per well. Cells were then placed in an incubator for 1 h at 35°C. After 1 h, cells were washed with 1× PBS and overlaid with a 1:1 mixture of DMEM (without FBS and PS) and Avicel, containing 2 µg/mL TPCK trypsin. Cells were placed in the incubator for 72 h at 35°C. After 72 h, the overlay was removed, cells washed with 1× PBS twice, and fixed with 2% PFA (in 1× PBS) for 20 min. After fixation, cells were later stained with 1% crystal violet for 20 min and washed twice. Plaques were visualized under the light, and PFU/mL was calculated, considering the serial dilution and initial volume for inoculation.

### IF analysis

After fixing with paraformaldehyde (4%) for 15 min, coverslips were washed three times with PBS for 5 min each. Cells were blocked and permeabilized with 0.1% Triton X-100 7% FBS in PBS for 1 h. Coverslips were washed three times with 0.01% Triton X-100 7% FBS in PBS for 5 min each. Primary antibody diluted in 0.01% Triton X-100, 7% FBS in PBS was added to coverslip overnight. The subsequent day, coverslips were washed with 0.01% Triton X-100 7% FBS in PBS for 5 min each. Secondary antibody diluted in 0.01% Triton X-100, 7% FBS in PBS was added to coverslips for 1 h. Coverslips were washed three times with 0.01% Triton X-100 7% FBS in PBS for 5 min each. Each coverslip was mounted with Dako mounting media containing 4′,6-diamidino-2-phenylindole (DAPI) and the respective organelle dyes unto glass microscope slides. Cytopainter Red ER staining kit (Abcam, UK, ab139482) was used to stain the ER according to the manufacturer's instructions. The Golgi was stained using GM130-AF647 (1:1000, Abcam, UK, ab195303). Coverslips were left overnight to dry. Slides were kept at 4°C until imaging. All images were acquired using Olympus FV3000RS confocal microscope. Images were acquired and deconvoluted using Imaris software (Olympus, Japan).

### Proximity ligation assay

To detect the association of Sec13 and influenza NS1 by proximity ligation assay (PLA), the Duolink *in situ* red starter kit was used (DUO92101-1KT—Sigma-Aldrich), following the manufacturer protocol exactly as described to perform this study.

### Immunoprecipitation

To each 10 cm dish, 500 µL of co-IP lysis buffer (20 mM Tris HCl pH 8, 137 mM NaCl, 1% NP-40, and 2 mM EDTA) with 1× phosphatase protease inhibitor was added and cells were scraped into 1.5 mL Eppendorf tubes. Tubes were kept on ice for 20 min and spun down at 10,000 g for 10 min at 4°C. Supernatant was transferred to new tubes and 12.5 µg of Anti-rabbit Dynabeads (Thermo Fisher Scientific) were added to pre-clear. Tubes were incubated on co-IP tube rotator for 30 min at room temperature with 10 rpm orbital speed. Tubes were then placed on a magnetic rack and the supernatant was transferred to a new t. Protein quantification was then conducted on the supernatant collected. Volumes equivalent to 1,000 µg of protein were aliquoted into new tubes and topped up with co-IP LB to a total volume of 500 µL. Four micrograms of antibodies were then added to 50 µg of Anti-rabbit Dynabeads and spun for 1 h on the co-IP rotator at room temperature. The tubes were then placed back onto the rotator in the cold room overnight. Next day, tubes were placed on the magnetic rack, supernatant was transferred to new tubes and 40 µg of protein samples were aliquoted into new tubes and boiled at 100°C for 5 min with loading dye. Pellets with beads were washed with co-IP lysis buffer by adding 250 µL to beads. Then, the tube was flicked to mix and placed on magnetic rack. This wash step was repeated three more times. After washing, 4 µL of 5× loading dye and 16 µL of lysis buffer were added to the beads and boiled at 95°C for 5 min. Samples were then loaded into Tris-glycine gels and ran as western blotting.

### Statistical tests

Manders overlap coefficient (MOC) analyses were performed for microscopy images. MOC measures the proportion of one protein colocalized with the other (21). All results are the mean ± SEM of 2–6 independent experiments. Student's *t*-test and one-way analysis of variance with Dunnett's multiple comparison tests were performed for all data sets. GraphPad Prism (v.7.0; GraphPad Software, La Jolla, CA, USA) was used for analysis and graph preparation. Values of *P* < 0.05 were considered statistically significant.

## RESULTS

### Molecular docking of Sec13 and NS1 reveal possible binding sites

As part of viral replication, viruses have to hijack various host organelles to transport and assemble new virions for export out of the cell. NS1 has been hypothesized to play an important role in influenza transmission and increased viral replication and viral pathogenicity (22, 23). We previously reported using *in silico* prediction that influenza proteins would bind to ER transport proteins, including Sec13 (5). A yeast screen was performed to identify novel host factors of influenza (Fig. S1) and it was observed that NS1 colocalized in the ER to Golgi vesicle (Sec13) but not late Golgi in yeast. Sec13 yeast and mammalian Sec13 are highly conserved (Fig. S2). In order to confirm the data, human Sec13 was docked with NS1 using ClusPro to predict the interaction of both proteins. As Sec13 is part of both the NPC and COPII complex, the crystal structure of Sec13 was part of a larger crystal structure of the NPC for PDB ID 3BG0 and 3BG1. This shows the interaction of NS1 with Sec13 at the NPC.

In order to understand the possible interaction of NS1 and Sec13, protein–protein docking was performed. It is a molecular technique that predicts the mutual orientation and position of two molecules forming a complex using computer algorithms and techniques. Molecular docking simulation was performed using the ClusPro web server (https://cluspro.bu.edu/login.php). ClusPro performs a ranking of docking models based on the size of the conformation cluster and provides two types of docking energies—the lowest energy among the conformations within a cluster of conformations and the core energy of the cluster (24). NS1 structures from different influenza strains, including an influenza B strain, as well as NS1 proteins from respiratory syncytial virus (RSV), dengue virus, and severe acute respiratory syndrome virus 2 (SARS-COV-2) were also included to characterize if this interaction was common among RNA viruses (Table S2).

The ClusPro score is calculated by attempting to find the native site with the lowest bind energy. Tables S2 and S3 show the size (number of members) of each cluster and the weighted energy score of the cluster center (i.e., the structure with most neighboring structures in the cluster) and the energy score of the lowest energy structure in the cluster (20). Based on the docking analysis, influenza NS1 and Sec13 can be docked with full-length NS1, RNA binding region (N-terminus), and effector domain (C-terminus). The ClusPro score ranged from −797.8 kJ/mol for influenza A/Brevig Mission/1/1918 (H1N1) NS1 RNA binding domain to −1047.6 kJ/mol for influenza A/Duck/Alberta/60/1976 (H12N5) NS1 effector domain with Sec13 (PDB ID: 3BG0, Table S2). This shows that there is no difference between NS1 from different influenza strains and whether the host was human or not. This would reflect a conserved interaction between NS1 and Sec13 as part of fundamental influenza virus biology.

Among the NS1 from different viruses, SARS-COV2 NSP1 had the highest ClusPro score of −1402.6 kJ/mol while RSV NS1 had the lowest ClusPro score of −800.9 kJ/mol with Sec13 (PDB ID: 3BG0, Table S2). This suggests a fundamental virus–host interaction among the RNA virus biology.

**Fig 1:**
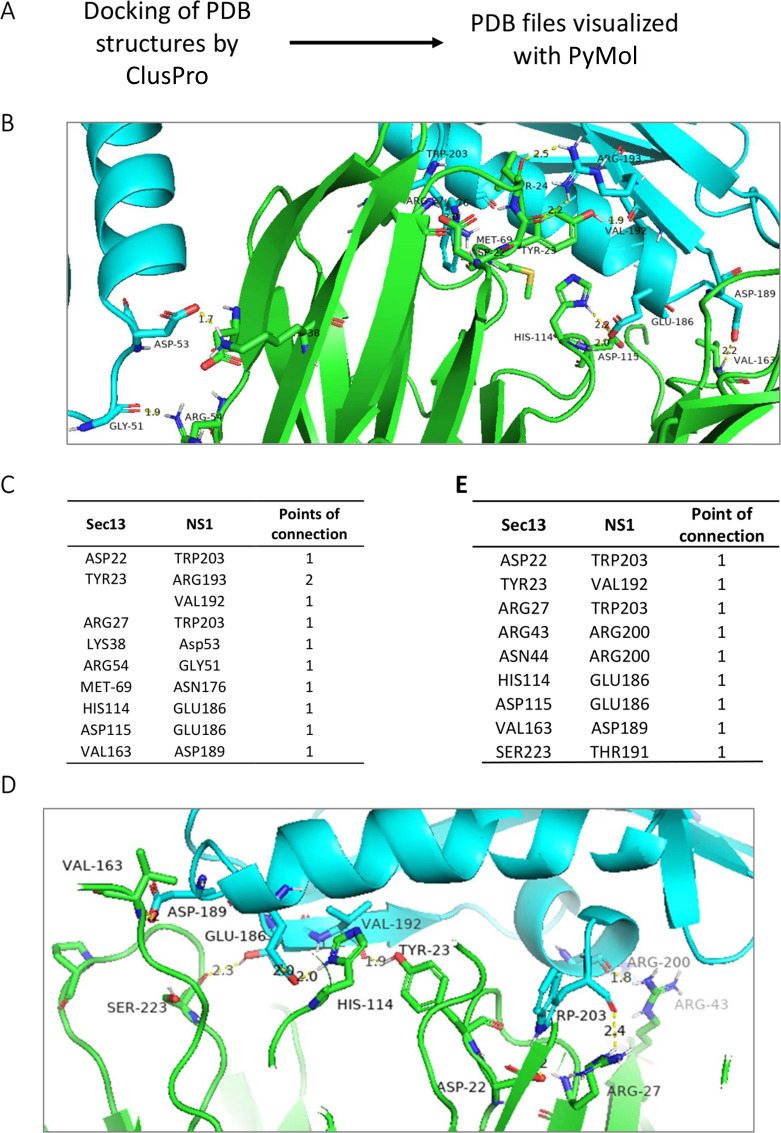
Docking representation of Sec13 and A/Puerto Rico/8/1934 (H1N1) NS1. (**A**) Workflow of docking analysis. (**B**) Visualization of the best-docked Sec13-NS1 complex in dimer form. Sec13 (PDB: 3BG0, chains ADEH) is green, while NS1 (PDB: 5NT2, chains CDEF) is blue. Docking analysis was performed with ClusPro 2.0. (**C**) Interacting residues of NS1 and Sec13 are shown in table. (**D**) Visualization of the best-docked Sec13-NS1 complex in dimer form. Sec13 (PDB: 3BG1, chains ADEH) is green, while NS1 (PDB: 5NT2, chains CDEF) is blue. Docking analysis was performed with ClusPro 2.

In order to further characterize the interaction with influenza NS1 and Sec13, the molecular docking complexes of NS1 (PDB ID: 5NT2) and Sec13 (PDB ID: 3BG0 and 3BG1), respectively, were used to analyze the amino acid interaction. The interaction was generated using PyMol (Fig. 1). It is observed that the C terminus of NS1 interacts more with Sec13 via TRP203, VAL192, GLU186, and ASP187.

### Sec13 co-stains and associates with NS1 during infection

**Fig 2:**
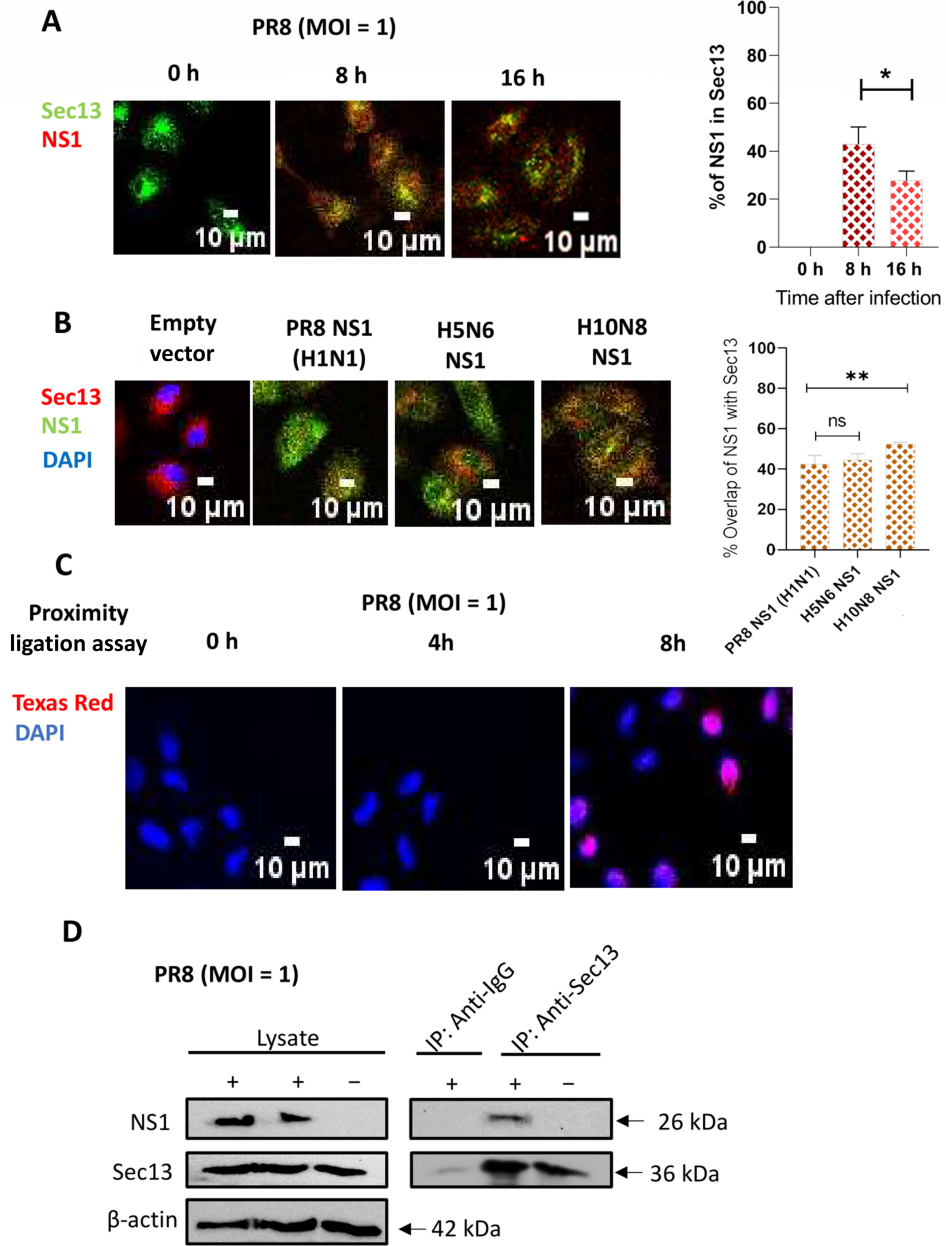
NS1 and Sec13 physically associate with each other in infected A549 cells. (**A**) A549 were seeded overnight on coverslips, infected with A/Puerto Rico/8/1934 (MOI = 1), fixed at various post-infection times, permeabilized, and stained with the NS1 and Sec13 antibodies. DAPI was used to stain the nucleus. Overlap percentage of NS1 with Sec13 using Manders overlap coefficient is graphed. (**B**) A549 cells were transfected with different NS1 plasmids for 16 h and fixed, permeabilized, and stained. % overlap of NS1 with Sec13 using Manders overlap coefficient is graphed. (**C**) Proximity ligation assay of A549 infected with A/Puerto Rico/8/1934 (MOI = 1) at different time points using NS1 and Sec13 antibodies. Scale is 10 µM. (**D**) Immunoprecipitation of A549 infected with A/Puerto Rico/8/1934 (MOI = 1) at 16 hpi lysates or uninfected lysates using anti-Sec13 antibody for pull-down. Infected A549 cell lysates were immunoprecipitated with IgG antibody as a negative control. Immunoblotting was performed with NS1 and Sec13 levels. β-actin was used as loading control for the whole cell extracts. Data are representative of *n* = 3 independent experiments.

We first confirmed the association of Sec13 with NS1 by performing immunostaining for influenza virus and Sec13 in influenza-infected cells. Influenza-infected cells were immunostained for Sec13 and NS1 to show infection efficiency and NS1-specific localization (Fig. 2A). Indeed, Sec13 was shown to colocalize with influenza NS1 protein after infection. Sec13 was in the cytoplasm throughout infection, localized outside the nucleus with NS1 at 8 h post-infection (hpi). At 16 hpi, the colocalization and association of NS1 and Sec13 reduced to ~25% (Fig. 2A). The decrease in NS1 and Sec13 overlap may be due to the change in NS1 and Sec13 localization during influenza.

We observed that overall Sec13 levels do not change during influenza infection and with different influenza strains (Fig. S3). Confocal microscopy of A549 cells transfected with plasmids encoding H1N1 PR8 NS1 and more pathogenic H5N6 NS1 and H10N8 NS1 as performed. Sec13 was observed mainly in the cytoplasm in control cells. When NS1 was transfected (Fig. 2B), Sec13 is observed again to colocalize with NS1.

H5N6 and H10N8 strains are avian influenza strains that have been found to infect humans. H5N6 is a highly pathogenic avian influenza virus strain isolated from a patient's throat swab specimen. The patient was exposed to poultry and developed primary viral pneumonia (25). H10N8 is a low pathogenic avian influenza strain and is the first known case of human infection. The patient developed severe pneumonia, septic shock, multi-organ failure, and succumbed on Day 9 (26). Higher co-staining of H10N8 NS1 with Sec13 was observed as compared with H1N1 NS1 (Fig. 2B). This data suggests that Sec13 may contribute to the pathogenicity of NS1, as the higher co-staining of NS1 and Sec13 corresponds to the degree of strain pathogenicity. The conservation of NS1 indicates the importance of Sec13 across different IAV strains, especially the pathogenic strains (Fig. S4).

A PLA was next performed to confirm the colocalization and to study the association of NS1 and Sec13. Figure 2C shows that NS1 and Sec13 are seen to be close to each other at 8 hpi, validating that NS1 and Sec13 interact during infection. To confirm this, an immunoprecipitation assay was performed with Sec13 pulldown in influenza-infected cells. Figure 2D shows that NS1 and Sec13 do associate and interact during influenza infection, confirming the docking results shown in Fig. 1.

### SEC13 and NS1 trafficking in ER and Golgi after influenza infection

**Fig 3:**
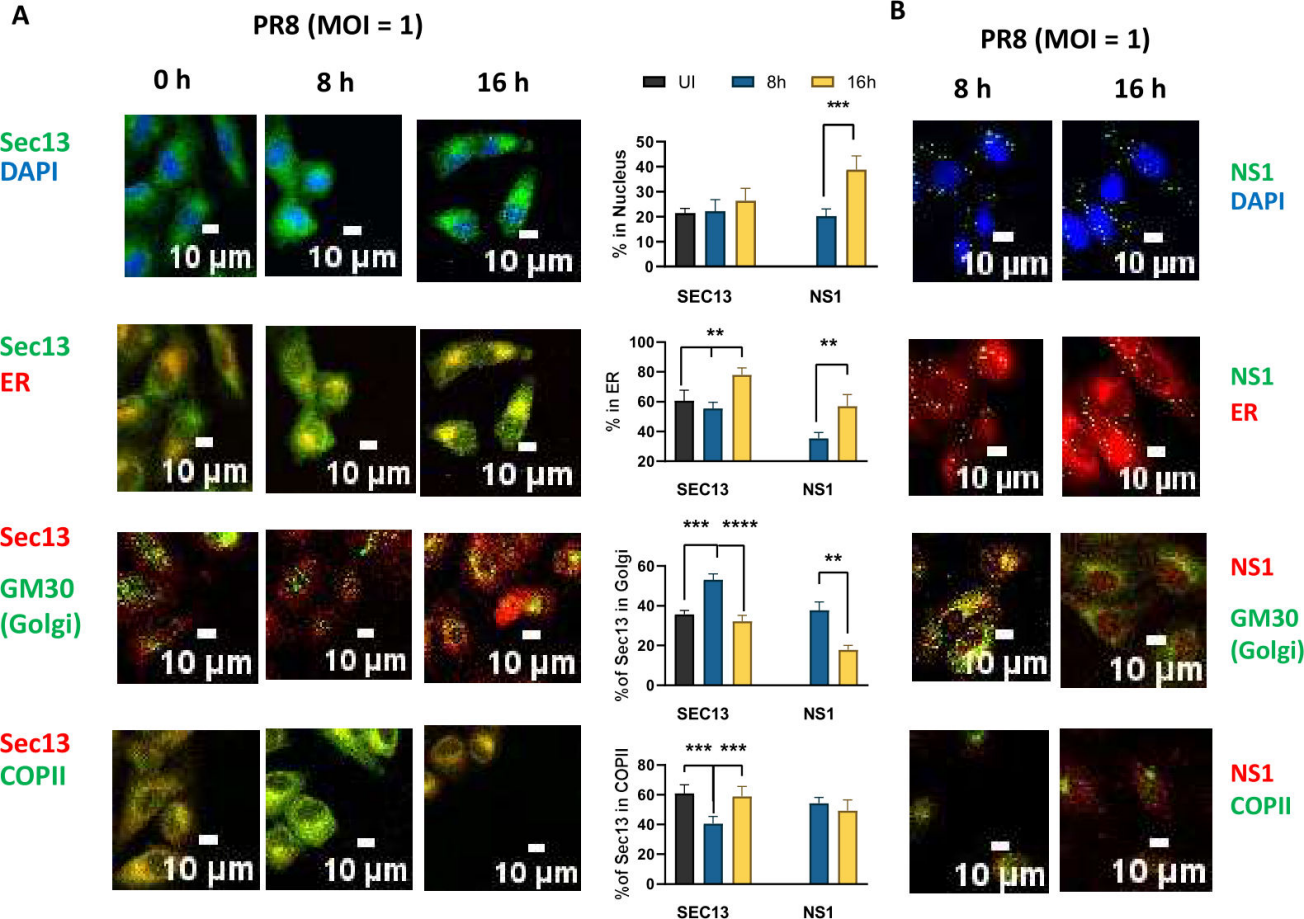
Sec13 and NS1 in various organelle compartments during influenza infection A549 cells were seeded on coverslips, infected with influenza virus for the indicated times and then fixed and permeabilized. Cells were immunostained with the respective antibodies. (**A**) Localization of Sec13 and DAPI, ER, GM130, and COPII (**B**) Localization of NS1 and DAPI, ER, GM130, and COPII Scale is 10 µM. Representative of three independent experiments.

Next, the localization of SEC13 was elucidated through immunofluorescence (IF) imaging of SEC13 and DAPI for nuclear staining as well as the ER, COPII vesicles, and the cis-Golgi (GM130). In uninfected cells, SEC13 was localized in the ER, in COPII vesicles (Sec23), and cis-Golgi and less in the nucleus.(Fig. 3) After 8h of infection, SEC13 did not increase in the nucleus but was more evident in the cis-Golgi and less in the COPII vesicles, while at 16 h, it was significantly enhanced in the ER. This indicates that SEC13 may dissociate from the COPII vesicles, redistributing to the Golgi at 8 h, and recycles back to the ER at 16 h.

Similarly, NS1 localization through the organelles was also evaluated. NS1 was found to be localized in the nucleus and cis-Golgi at 8 h, and is transported to the ER and the nucleus, out of the cis-Golgi at 16h (Fig. 3C). The similar trafficking of Sec13 and NS1 in the Golgi to ER at 8 and 16 h, respectively, and that NS1 and Sec13 interac could imply that NS1 binds to Sec13 during this redistribution of NS1 from the Golgi to the ER at later time points of infection.

### Inhibition of ER-to-Golgi transport and Sec13 regulation affected viral titers

**Fig 4:**
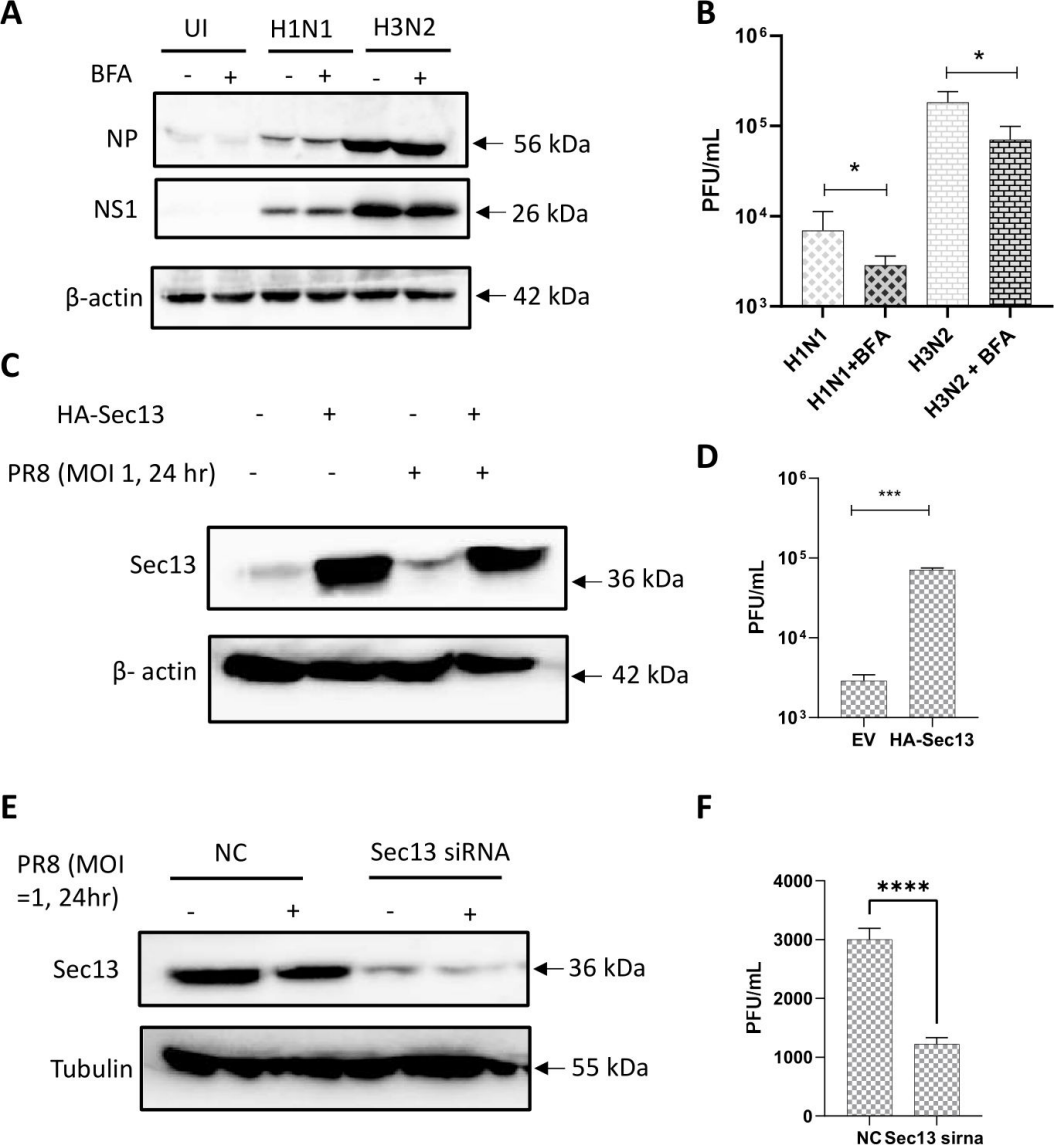
Effect of inhibition of ER to Golgi pathway and Sec13 regulation on viral titers in A549 cells. A549 cells were pre-treated with 5 µg/mL BFA for 2 h and then infected with H1N1 or H3N2 (MOI = 1) at 24 hpi. Cells and supernatant were then harvested and assessed for (**A**) viral proteins using western blot (**B**) plaque assay for virus titer. A549 cells were transfected with EV and HA-Sec13 plasmids overnight and then infected with H1N1 (MOI 1) at 24 hpi. Cells and supernatant were then harvested and assessed for (**C**) viral proteins using western blot (**D**) plaque assay for virus titer. A549 cells were transfected with 10 nM NC or Sec13 siRNA overnight and then infected with H1N1 (MOI 1) at 24 hpi. Cells and supernatant were then harvested and assessed for (**E**) viral proteins using western blot (**F**) plaque assay for virus titer. Data is representative of mean ± SEM of 3–5 independent experiments. Hpi: hours post-infection. EV: empty vector. NC: negative control. *P < 0.05; ***P < 0.001; ****P ≤ 0.0001 versus controls (Control, EV, NC).

Brefeldin A (BFA) was used to investigate the effect of ER-Golgi transport inhibition on influenza viral titers. BFA-treated cells exhibited increased NS1 and NP expression at 24 hpi (Fig. 4A) but reduced virus plaque formation with the respective IAV strains (Fig. 4B). This implies that inhibition of ER-Golgi transport resulted in accumulation of viruses in the cells, affecting the intracellular transport of viral proteins to the cell membrane, resulting in decreased viral budding out of the cell.

Next, Sec13 was overexpressed and silenced using siRNA. Sec13 overexpression (Fig. 4C) resulted in an increase in virus plaque formation (Fig. 4D) compared to the empty vector (EV) transfected cells. In contrast, Sec13 knockdown (Fig. 4E) reduced viral plaque formation compared to NC cells (Fig. 4F).

### Sec13 knockdown decreased NS1 localization in ER and Golgi

**Fig 5:**
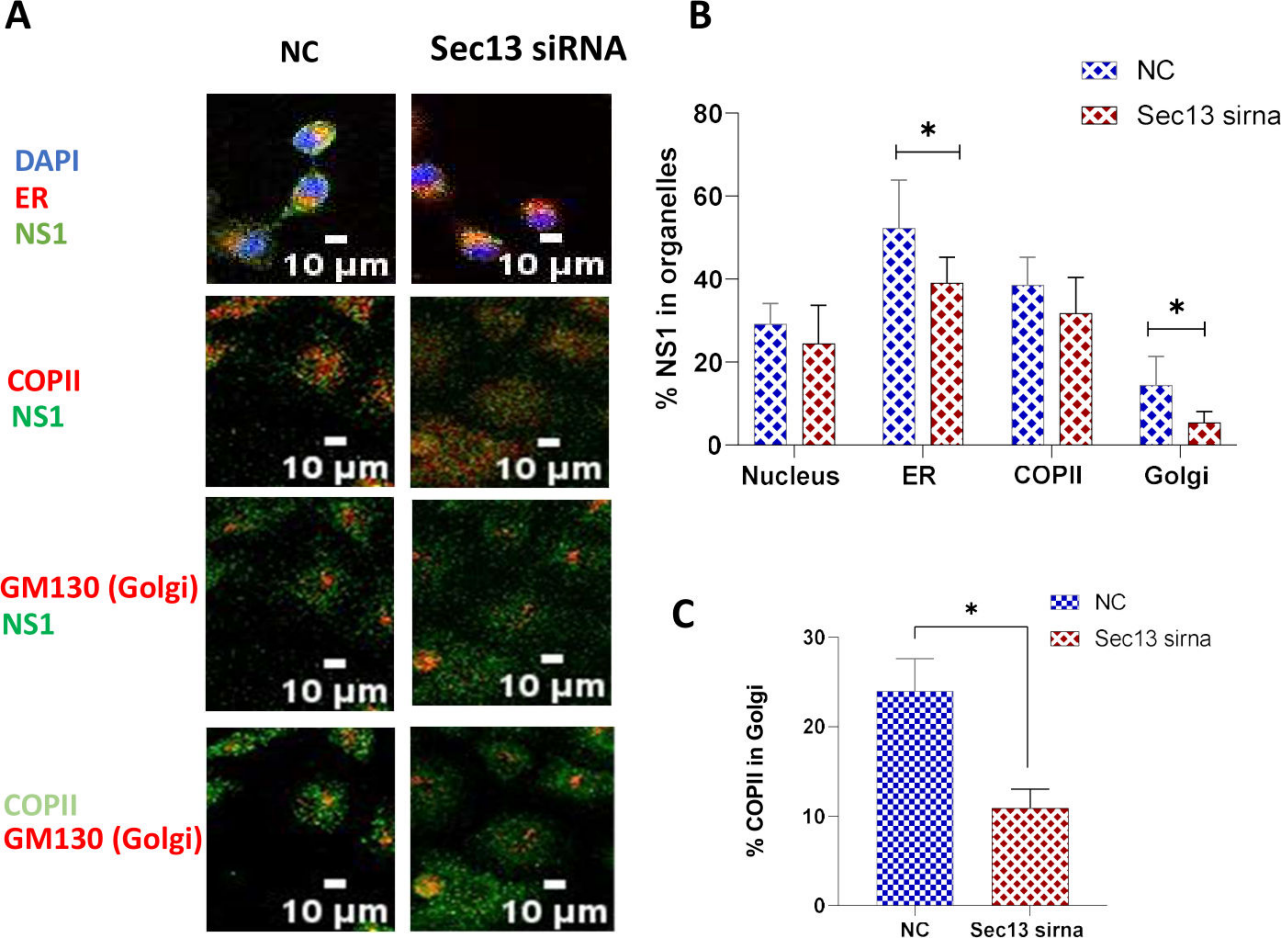
Localization of NS1 and COPII in Sec13 knockdown infected cells. (**A**) A549 cells were seeded on coverslips and then transfected with 10 nM NC or Sec13 siRNA overnight, followed by infected with H1N1 (MOI = 1) were fixed at 16 hpi, permeabilized, and stained with NS1, COPII, and GM130 antibodies, respectively. DAPI was used to stain the nucleus. Cytopainter Red ER staining kit was used to stain the ER. (**B**) MOC percentage of NS1 overlap with various organelles in NC or Sec13 siRNA infected cells. NC: Negative control. Hpi: hours post-infection. Scale is 10 µM. Data is representative of mean ± SEM from nine replicates of three independent experiments. *P < 0.05; **P < 0.01 versus NC sample.

Control or Sec13 siRNA transfected influenza-infected cells were immunostained for NS1 and the various organelles (Fig. 5A; Fig. S5). Sec13 silencing did not affect nuclear localization of NS1, confirming that Sec13 does not play a role in the nuclear transport of NS1. However, there was less NS1 detected in the ER and cis-Golgi (Fig. 5B) when Sec13 was silenced, with no change in the COPII complex association. This decreased NS1 association with Golgi was also correlated with a decreased association of COPII with the Golgi (Fig. 5C). Together, these results reflect that reduced levels of Sec13 results in impaired ER-to-Golgi transport, which in turn, may affect viral budding (Fig. 4F).

## DISCUSSION

In the present study, we identified and validated Sec13 a novel host interactor of influenza. We showed that Sec13 is associated with ER, COPII, and Golgi in infected lung epithelial cells and not the nucleus during PR8 infection. Colocalization of NS1 and Sec13 were correlated at several time points of infection and inhibiting the ER-to-Golgi transport and silencing Sec13 decreased viral titers, whereas overexpressing Sec13 increased viral titers. Hence, we propose that the ER-to-Golgi transport is an important pathway of viral replication and viral export, and specifically, Sec13 has a functional role in influenza replication and virulence.

Sec13 has been previously identified as an essential host interactor in human HIV (19) and plant BMV (18) viral infections. Namely, localization of BMV1a to the perinuclear ER is disrupted when cells express dysfunctional Sec13 (18). In addition, silencing of SEC13 inhibits HIV-1 infection by reducing HIV integration (19). This shows evidence of an important role of Sec13 in viral replication (27). Sec13 was also identified as a host interactor of influenza in a CRISPR knockout screen (25). Our docking analysis confirms this and that the C terminus of NS1 may interact more with Sec13. This would imply the effector domain of NS1 is more important in the interaction with Sec13 and could be related to downstream functions of NS1. This observation is interesting as these residues are closer to the nuclear localization signal of NS1, which is found in the C-terminal region (26, 28) and the nuclear export signal (29). Given that the RNA binding domain of NS1 could also be docked with Sec13 (Table S2), this could be related to the pathogenicity of different influenza strains. Key amino acid mutations in the RNA binding domain of NS1 have been identified to increase pathogenicity, along with crucial interactions with host proteins (22, 28). Given the proposed interaction between NS1 and Sec13 at the RNA binding domain, this underscores the significance of Sec13 in viral pathogenicity.

Our confirmation of NS1 binding and association with Sec13 using PLA and immunoprecipitation shows that NS1 binds to Sec13 for its transport and life-cycle, and subsequent regulation of effectors in the ER. Several post-translation modifications, such as phosphorylation, SUMOlyation, and ISGylation which occur in the ER have been identified on NS1 (30). Such post-translational modifications have been described to play critical roles in NS1 function and phosphorylation of NS1 at different residues has been shown to affect its activity (31). Moreover, SUMOlyation of NS1 is required for its activity but too little or too much SUMOlyation affects its interferon-blocking functions. However, the host can use post-translational modification against NS1 by ISGylating it to limit its activity (32, 33). Moreover, NS1 itself affects the post-translational modifications of multiple host proteins such as AKT, p53, TRIM25, and RIG-I (31). NS1 has been shown to interact with RAP55-associated *P* bodies, stress granules, and cytoplasmic RNA granules (34). Moreover, NS1 was shown to be a key modulator of ER stress during influenza infection (35). Interestingly, our novel finding that NS1 was also identified in the COPII vesicles and Golgi, indicates NS1 in ER to Golgi transport. This result is interesting because NS1 is not typically found in packaged virions (36). To the author's current knowledge, NS1 has not been previously reported in the ER-to-Golgi transport or other vesicle transport.

This finding would correlate with the importance of ER-to-Golgi transport for STING translocation in influenza. Translocation of STING to the Golgi is required to assemble with TBK1 to induce interferon production (37). Influenza has been shown to interact with STING via the hemagglutinin (HA) fusion protein (38). This interaction inhibits STING association with TBK1 and downstream interferon production (38). This information would imply an additional role for NS1 in affecting the STING pathway, given its role in inhibiting interferon production. ZIKV NS1 thwarts the cGAS pathway indirectly by enlisting the host enzyme ubiquitin-specific protease 8 (USP8). USP8 targets and cleaves K11-linked poly-ubiquitin chains from L134, thus preventing the proteasomal degradation of caspase-1. The stabilized caspase-1 subsequently enhances the cleavage of cGAS, leading to its activation (39). Similarly, ZIKV also antagonizes cGAS activity, albeit indirectly, by promoting the stabilization of caspase-1 through NS1. This stabilized caspase-1, in turn, cleaves and activates cGAS (40). In the case of IAV, the NS1 protein has a dual role in inhibiting cGAS signaling. First, it binds to and sequesters mitochondrial DNA (mtDNA) within infected cells, reducing cGAS detection of mis-localized DNA. Additionally, IAV's HA protein interferes with STING dimerization and the subsequent activation of TBK1. Notably, IAV's NS1 protein has been found to directly interact with mtDNA, reducing the activation of cGAS-dependent antiviral responses (41).

Sec13 was overexpressed to study the role of Sec13 in influenza infection. Sec13 overexpression increased viral titers while silencing Sec13 decreased viral titers (Fig. 4). This result reflects the importance of Sec13 in influenza replication and the innate immune response and confirms other results, where Sec13 was described as a component in VISA-mediated antiviral signaling, and Sec13 knockdown decreased IFNβ expression (42). Sec13 silencing reduced the localization of NS1 in the ER and Golgi.

From the Sec13 localization time course during influenza infection (Fig. 4), Sec13 was not observed to be localized in the nucleus and was instead more colocalized in the ER, COPII vesicles (Sec23), and cis-Golgi in uninfected cells. This observation suggests greater Sec13 involvement in the ER and Golgi, possibly in ER-to-Golgi transport. After 8 h of infection, less Sec13 was observed at the COPII vesicles, while more Sec13 were found in the cis-Golgi compared to uninfected cells. This accumulation may be due to a redistribution of Sec13 to the Golgi via COPII during early infection. This observation highlights the importance of COPII transport in the early stages of influenza infection. At 16 hpi, representing a late infection time point toward the end of virus replication cycle, Sec13 was observed to localize more with the ER and COPII complex. This observation would imply recycling of Sec13 back to the ER from the Golgi at later time points. Once Sec13 is recycled back to ER, this would enable more Sec13 to enter the COPII complex. In the case of NS1, more association was observed in the COPII vesicles and cis-Golgi at 8 hpi, mirroring that of Sec13. More NS1 is observed to enter the ER and nucleus at 16 hpi. Thus, these results may imply that both Sec13 and NS1 accumulate in the Golgi at early infection, exiting the Golgi and entering the ER at late infection. When Sec13 was silenced and cells were infected for 16 h, less NS1 was observed in the ER and cis-Golgi, and the association of the COPII complex with the Golgi was also lower. Given that Sec13 is on the outer coat of the COPII complex, this may impact the association of COPII vesicles to the cis-Golgi.

**Fig 6:**
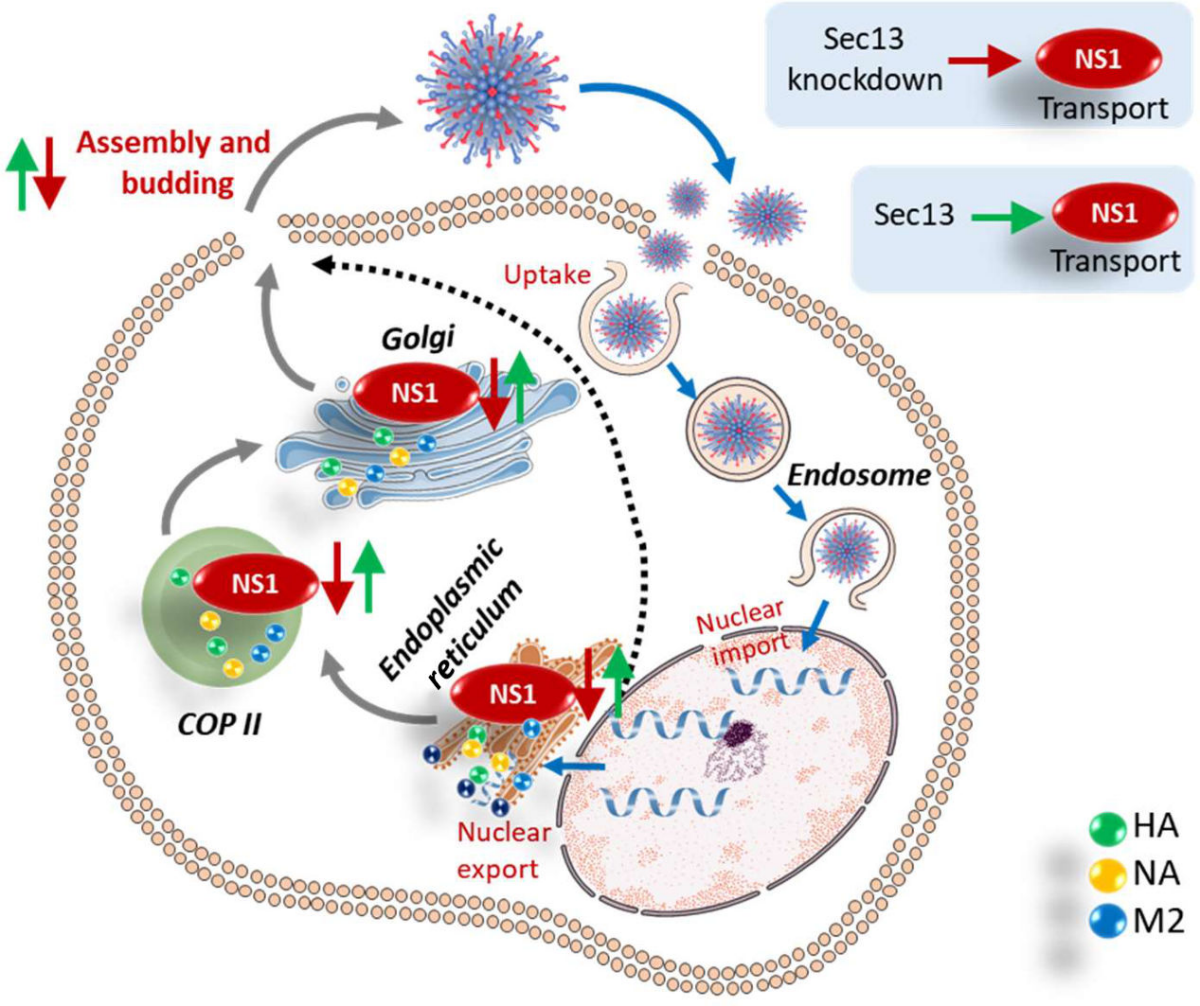
Schematic representation of Sec13 knockdown on influenza infection in A549 lung epithelial cells. During infection, the translocation of Sec13 and NS1 was shown in the ER; COPII vesicles and Golgi are essential for viral replication. Sec13 knockdown resulted in decreased NS1 localization in the ER and Golgi. Hence, there was decreased viral budding out of the cell. Hpi: Hours post-infection. The arrow in red and green indicates the role of suppression and promotion respectively.

The proposed mechanism is seen in Fig. 6. Hence, NS1 requires Sec13 for transport from the ER to the Golgi during viral replication. This confirms the results observed in our previous bioinformatics analysis report (5), which described that many host interactors of influenza were involved in protein processing in the ER.

It is essential to understand the critical host processes that influenza targets and hijacks the development of host-directed therapy, as host processes are less likely to develop resistance due to viral mutations. Hence, Sec13 has been identified as a novel host interactor and plays an essential role in the ER-to-Golgi pathway during influenza infection.

As influenza is an enveloped virus, this data may also apply to other enveloped viruses. This can be seen by docking analysis in Tables S1 to S3. Sec13 was also identified as a host interactor of HIV via a multiple orthologous RNAi reagents screen (19). Given that it has been shown that Sec13 is mainly associated with COPII vesicles during IAV infection, this is similar to other studies with enveloped viruses such as Sendai virus (43), Chikungunya virus (44), and Ebola virus (45). In addition, the importance of the COPII pathway in non-enveloped viruses such as poliovirus has also been described (46). Hence, the ER-to-Golgi pathway is an important pathway manipulated by viruses for their replication. This may serve as a potential therapeutic target in viral disease.

## SUPPLEMENTAL MATERIAL

Supplemental material spectrum.02609-23-s0001.docx Tables S1 to S3 and Fig. S1 to S4.

ASM does not own the copyrights to Supplemental Material that may be linked to, or accessed through, an article. The authors have granted ASM a non-exclusive, world-wide license to publish the Supplemental Material files. Please contact the corresponding author directly for reuse.
