# Supplementary material for: The ER-Golgi transport of influenza virus through NS1-Sec13 association during virus replication
Source: Microbiol Spectr. 2023 Dec 1;12(1):e02609-23. doi: 10.1128/spectrum.02609-23 (PMC10782970; doi:10.1128/spectrum.02609-23)
Supplement: Supplemental material — Tables S1 to S3 and Fig. S1 to S4. [file spectrum.02609-23-s0001.docx]

Supplementary table S1. List of PDB files used for CLUSPRO analysis

| PDB ID | | Virus (Strain) | Link |
| --- | --- | --- | --- |
| 3RVC | Influenza A (A/Puerto Rico/8/1934 (H1N1)) | | https://www.rcsb.org/structure/3rvc |
| 5NT2 | Influenza A (A/Puerto Rico/8/1934 (H1N1)) | | https://www.rcsb.org/structure/5nt2 |
| 2N74 | Influenza A virus (A/Brevig Mission/1/1918(H1N1)) | | https://www.rcsb.org/structure/2n74 |
| 3D6R | Influenza A (A/duck/Alberta/60/1976(H12N5)) | | https://www.rcsb.org/structure/3d6r |
| 3EE8 | Influenza A (A/Udorn/307/1972(H3N2)) | | https://www.rcsb.org/structure/3ee8 |
| 4O6B | Dengue virus (Dengue 2/Thailand/16681/84) | | https://www.rcsb.org/structure/406b |
| 5BXZ | Influenza A (A/little yellow-shouldered bat/Guatemala/153/2009(H17N10)) | | https://www.rcsb.org/structure/5bxz |
| 5BY1 | Influenza A (A/flat-faced bat/Peru/033/2010(H18N11)) | | https://www.rcsb.org/structure/5by1 |
| 5VJ2 | Respiratory syncytial virus type A | | https://www.rcsb.org/structure/5vj2 |
| 4OIG | Dengue virus (Dengue virus 1 Nauru/West Pac/1974) | | https://www.rcsb.org/structure/40ig |
| 3RT3 | Influenza B virus (B/Lee/1940) | | https://www.rcsb.org/structure/3rt3 |
| 6ZOJ | Severe acute respiratory syndrome coronavirus 2 | | https://www.rcsb.org/structure/6zoj |

Supplementary Table S2. Weighted scores of the interaction of each NS1 structure with Sec13 (PDB ID: 3BG0)

| PDB | RBD | Cluster | Members (docked conformations) | Representative |
| --- | --- | --- | --- | --- |
| 3RT3 | Influenza B/Lee/1940 NS1 | 0 | 123 | Center |
|  |  |  |  | Lowest Energy |
| 5NT2 | Influenza A/Puerto Rico/8/34 (PR8) NS1 | 0 | 32 | Center |
|  |  |  |  | Lowest Energy |
| 5VJ2 | Respiratory Syncytial Virus (RSV) A NS1 | 0 | 32 | Center |
|  |  |  |  | Lowest Energy |
| 4OIG | Dengue virus 1 Nauru/West Pac/1974 NS1 | 0 | 23 | Center |
|  |  |  |  | Lowest Energy |
| 4O6B | Dengue virus 2 Thailand/16681/84 NS1 | 0 | 43 | Center |
|  |  |  |  | Lowest Energy |
| 2N74 | Influenza A/Brevig Mission/1/1918 (H1N1) NS1 RNA binding domain | 0 | 38 | Center |
|  |  |  |  | Lowest Energy |
| 3D6R | Influenza A/Duck/Alberta/60/1976 (H12N5) NS1 effector domain | 0 | 61 | Center |
|  |  |  |  | Lowest Energy |
| 5BY1 | Influenza A/Flat-faced bat/Peru/033/2010 (H18N11) NS1 RNA binding domain | 0 | 80 | Center |
|  |  |  |  | Lowest Energy |
| 5BXZ | Influenza A/Little yellow-shouldered bat/Guatemala/153/2009 (H17N10) NS1 RNA binding domain | 0 | 58 | Center |
|  |  |  |  | Lowest Energy |
| 3EE8 | Influenza A/Udorn/307/1972 (H3N2) Effector Domain | 0 | 64 | Center |
|  |  |  |  | Lowest Energy |
| 3RVC | Influenza A/Puerto Rico/8/34 (PR8) NS1 Effector domain | 0 | 66 | Center |
|  |  |  |  | Lowest Energy |
| 6ZOJ | Severe acute respiratory syndrome coronavirus 2 (SARS-COV-2) NSP1 | 0 | 75 | Center |
|  |  |  |  | Lowest Energy |

Supplementary Table S3. Weighted scores of the interaction of each NS1 structure with Sec13 (PDB ID: 3BG1)

| PDB | RBD | Cluster | Members (docked conformations) | Representative | Weighted score KJ/mol |
| --- | --- | --- | --- | --- | --- |
| 3RT3 | Influenza B/Lee/1940 NS1 | 0 | 78 | Center | -762.6 |
|  |  |  |  | Lowest Energy | -871.7 |
| 5NT2 | Influenza A/Puerto Rico/8/34 (PR8) NS1 | 0 | 24 | Center | -884.8 |
|  |  |  |  | Lowest Energy | -884.8 |
| 5VJ2 | RSV A NS1 | 0 | 42 | Center | -803.6 |
|  |  |  |  | Lowest Energy | -803.6 |
| 4OIG | Dengue virus 1 Nauru/West Pac/1974 NS1 | 1 | 25 | Center | -1033.1 |
|  |  |  |  | Lowest Energy | -1033.1 |
| 4O6B | Dengue virus 2 Thailand/16681/84 NS1 | 0 | 28 | Center | -946.2 |
|  |  |  |  | Lowest Energy | -1045.7 |
| 2N74 | Influenza A/Brevig Mission/1/1918 (H1N1) NS1 effector domain | 1 | 33 | Center | -753.1 |
|  |  |  |  | Lowest Energy | -753.1 |
| 3D6R | Influenza A/Duck/Alberta/60/1976 (H12N5) NS1 effector domain | 0 | 60 | Center | -1002.9 |
|  |  |  |  | Lowest Energy | -1037.1 |
| 5BY1 | Influenza A/Flat-faced bat/Peru/033/2010 (H18N11) RNA binding domain | 0 | 50 | Center | -665.9 |
|  |  |  |  | Lowest Energy | -852.8 |
| 5BXZ | Influenza A/Little yellow-shouldered bat/Guatemala/153/2009 (H17N10) RNA binding domain | 0 | 64 | Center | -739.4 |
|  |  |  |  | Lowest Energy | -773 |
| 3EE8 | Influenza A/Udorn/307/1972 (H3N2) Effector Domain | 0 | 46 | Center | -834.9 |
|  |  |  |  | Lowest Energy | -895.2 |
| 3RVC | Influenza A/Puerto Rico/8/34 (PR8) NS1 Effector domain | 0 | 54 | Center | -826.2 |
|  |  |  |  | Lowest Energy | -841.6 |
| 6ZOJ | Severe acute respiratory syndrome coronavirus 2 NSP1 | 1 | 45 | Center | -1177.2 |
|  |  |  |  | Lowest Energy | -1303 |

**Supplementary Figures**


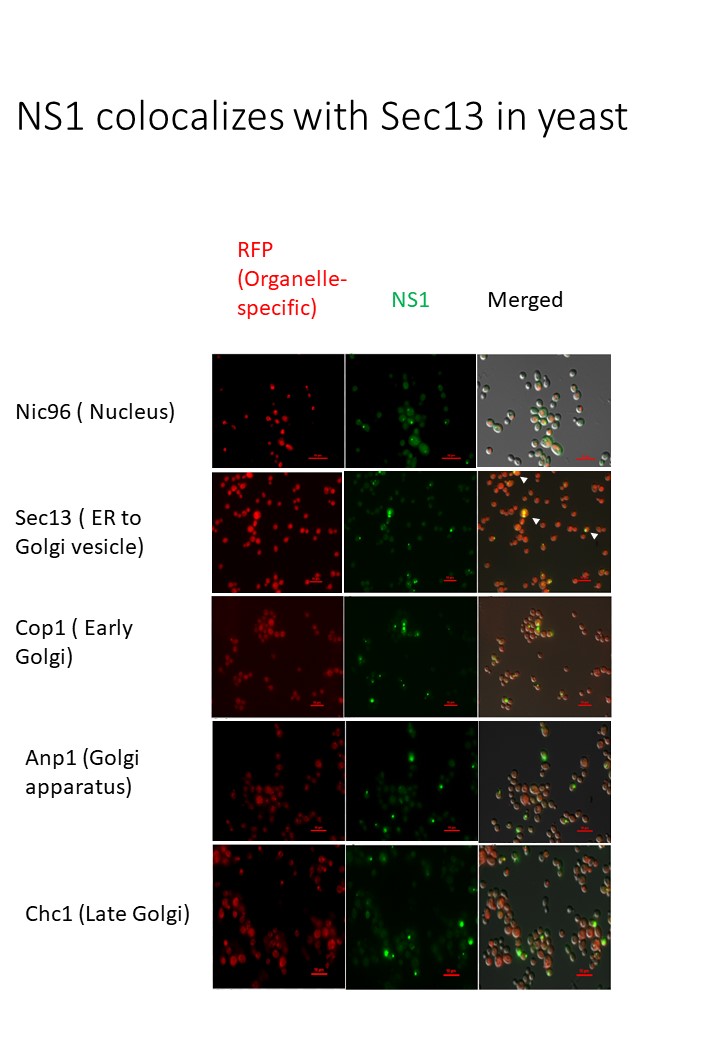


**Supplementary Figure 1:** **NS1 colocalizes with Sec13 in yeast.** Log phase yeast expressing RFP tagged organelles and NS1 tagged with GFP were examined under the microscope. Scale is 10 µM—representative of 3 independent experiments.


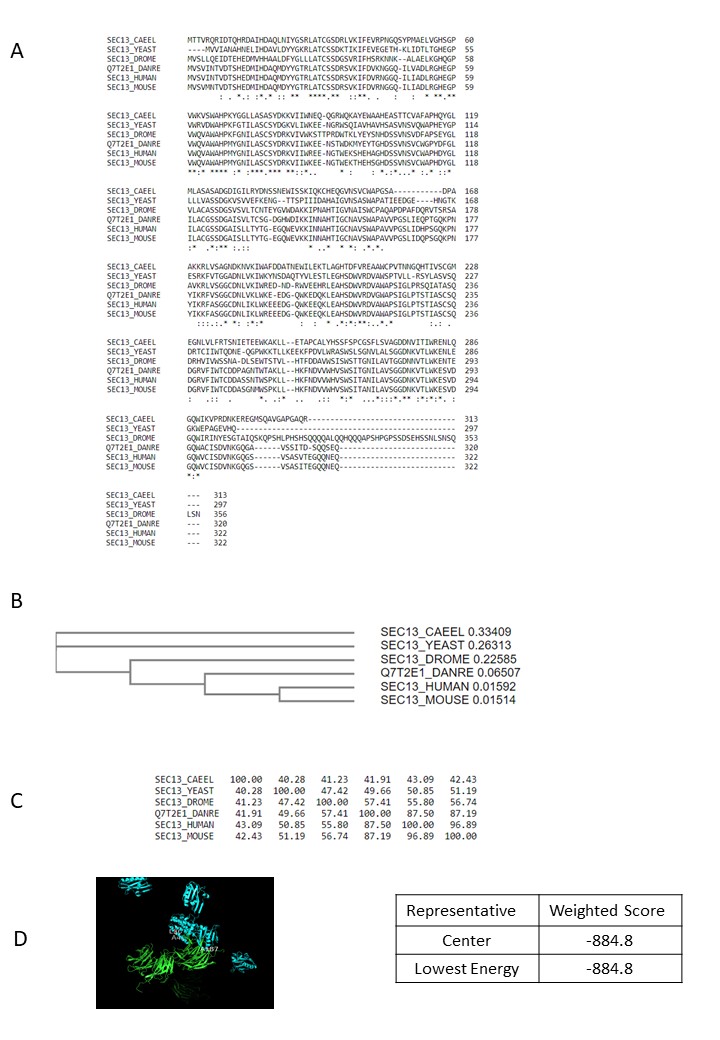


**Supplementary Figure 2:** **Sec13 is conserved across the various species.** (A) MSA of human Sec13 with Sec13 from other species. An * (asterisk) indicates positions with a single, fully conserved residue. A : (colon) indicates conservation between groups of strongly similar properties as below - roughly equivalent to scoring > 0.5 in the Gonnet PAM 250 matrix. A . (period) indicates conservation between groups of weakly similar properties as below - roughly equivalent to scoring =< 0.5 and > 0 in the Gonnet PAM 250 matrix. (B) Phylogenetic tree of the various Sec13 (C) Amino Acid Percentage similarity of the various Sec13. All analysis was performed by Clustal2.1. (D) Visualization of the best-docked Sec13-NS1 complex in dimer form. Sec13 (PDB: 3BG1, chains ADEH) is green, while NS1 (PDB: 5NT2, chains CDEF) is blue. Docking analysis was performed with CLUSPRO 2.0. Key residues are highlighted in red.

**Supplementary Figure 3: Sec13 levels in influenza infected cells of different strain, multiplicity of infection (M.O.I.) and timepoint.** A549 cells were infected with H1N1 (A/Puerto Rico/8/1934) or H3N2 at various M.O.I. Cells and supernatant were then harvested and assessed for Sec13 and NS1 using western blot. (A) H1N1 infection at 24 hpi, (B) H1N1 infection at 48 hpi, (C) H3N2 infection at 24 hpi, (C) H3N2 infection at 48 hpi. Hpi: hours post infection.


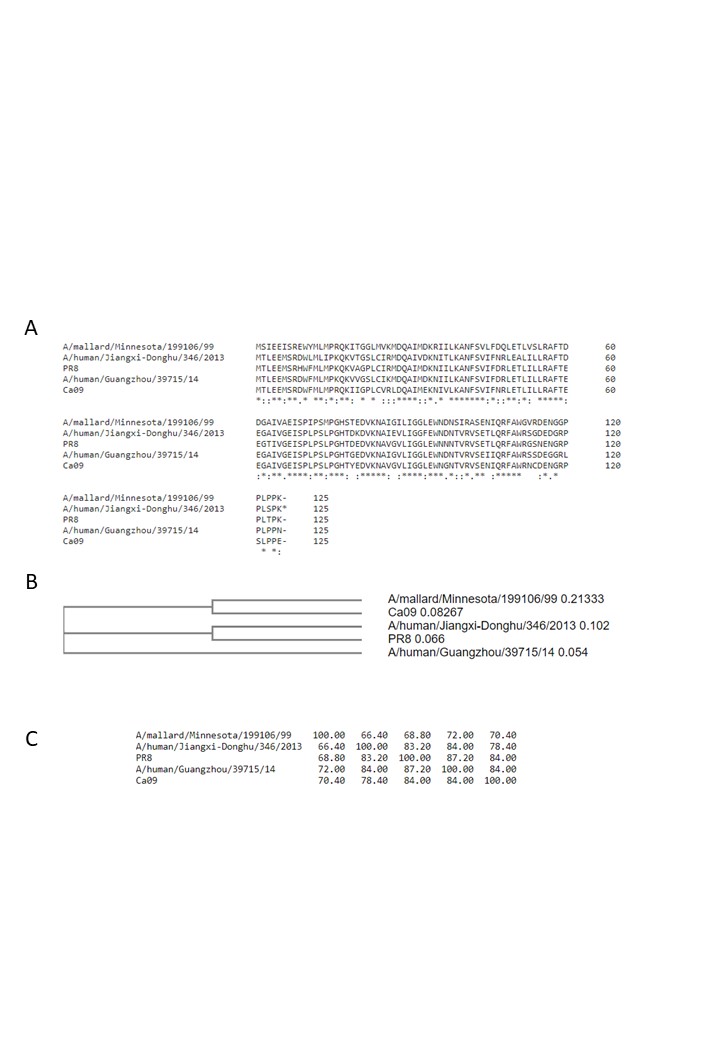


**Supplementary Figure 4** **NS1 is conserved across the various species.** **(A)** MSA of NS1 different influenza strains. An * (asterisk) indicates positions with a single, fully conserved residue. A : (colon) indicates conservation between groups of strongly similar properties as below - roughly equivalent to scoring > 0.5 in the Gonnet PAM 250 matrix. A . (period) indicates conservation between groups of weakly similar properties as below - roughly equivalent to scoring =< 0.5 and > 0 in the Gonnet PAM 250 matrix. **(B)** Phylogenetic tree of the various NS1 **(C)** Amino Acid Percentage similarity of the different NS1. All analysis was performed by Clustal2.1. PR8: A/Puerto Rico/8/1934 (H1N1)
